# A Growth Differentiation Factor 15-Based Risk Score Model to Predict Mortality in Hemodialysis Patients

**DOI:** 10.3390/diagnostics11020286

**Published:** 2021-02-11

**Authors:** Jia-Feng Chang, Po-Cheng Chen, Chih-Yu Hsieh, Jian-Chiun Liou

**Affiliations:** 1 Division of Nephrology, Department of Internal Medicine, En Chu Kong Hospital, New Taipei City 237, Taiwan; cjf6699@gmail.com (J.-F.C.); fish37435@hotmail.com (C.-Y.H.); 2 Department of Nursing, Yuanpei University of Medical Technology, Hsinchu 300, Taiwan; 3 Division of Nephrology, Department of Internal Medicine, Shuang Ho Hospital, Taipei Medical University, New Taipei City 235, Taiwan; 4 Graduate Institute of Aerospace and Undersea Medicine, Department of Medicine, National Defense Medical Center, Taipei 114, Taiwan; 5 Renal Care Joint Foundation, New Taipei City 220, Taiwan; 6 Department of Urology, En Chu Kong Hospital, New Taipei City 237, Taiwan; b90401049@ntu.edu.tw; 7 School of Biomedical Engineering, Taipei Medical University, Taipei 110, Taiwan

**Keywords:** growth differentiation factor 15, death prediction model, hemodialysis

## Abstract

Background: The risk of cardiovascular (CV) and fatal events remains extremely high in patients with maintenance hemodialysis (MHD), and the growth differentiation factor 15 (GDF15) has emerged as a valid risk stratification biomarker. We aimed to develop a GDF15-based risk score as a death prediction model for MHD patients. Methods: Age, biomarker levels, and clinical parameters were evaluated at study entry. One hundred and seventy patients with complete information were finally included for data analysis. We performed the Cox regression analysis of various prognostic factors for mortality. Then, age, GDF15, and robust clinical predictors were included as a risk score model to assess the predictive accuracy for all-cause and CV death in the receiver operating characteristic (ROC) curve analysis. Results: Age, GDF15, and albumin were significantly associated with higher all-cause and CV mortality risk that were combined as a risk score model. The highest tertile of GDF-15 (>1707.1 pg/mL) was associated with all-cause mortality (adjusted hazard ratios (aHRs): 3.06 (95% confidence interval (CI): 1.20–7.82), *p* < 0.05) and CV mortality (aHRs: 3.11 (95% CI: 1.02–9.50), *p* < 0.05). The ROC analysis of GDF-15 tertiles for all-cause and CV mortality showed 0.68 (95% CI = 0.59 to 0.77) and 0.68 (95% CI = 0.58 to 0.79), respectively. By contrast, the GDF15-based prediction model for all-cause and CV mortality showed 0.75 (95% CI: 0.67–0.82) and 0.72 (95% CI: 0.63–0.81), respectively. Conclusion: Age, GDF15, and hypoalbuminemia predict all-cause and CV death in MHD patients, yet a combination scoring system provides more robust predictive powers. An elevated GDF15-based risk score warns clinicians to determine an appropriate intervention in advance. In light of this, the GDF15-based death prediction model could be developed in the artificial intelligence-based precision medicine.

## 1. Introduction

Patients receiving maintenance hemodialysis (MHD) have an exceedingly high risk of fatal events, and cardiovascular diseases (CVD) still top list of death causes [1]. Persistent, low-grade inflammation accompanied by malnutrition acts as a hallmark feature of chronic kidney disease (CKD), being involved in the pathogenesis of CVD and the development of all-cause mortality of MHD patients [2,3]. Growth differentiation factor-15 (GDF15), also known as macrophage inhibitory cytokine-1 belonging to the transforming growth factor β superfamily, is a stress-induced protein [4]. In general population, circulating levels of GDF15 are highly elevated in response to pathological conditions such as oxidative stress, inflammation, tissue injury, metabolic dysregulation, autoimmune diseases, cancer entities, and hypoxia-related cardiovascular (CV) ailments [5]. In CKD patients, GDF15 levels are notably raised and responsible for the greater risks of CKD progression, CV morbidity, and mortality [6,7,8,9,10].

Whilst much has been done to better understand risk factors for fatal events in CKD patients, novel biomarkers and conventional risk stratification tools do not adequately predict prognosis and prevent mortality. Accordingly, fatality remains alarmingly high in CKD despite global age-standardized mortality being decreased by 30.4% for CVD, 14.9% for cancer and 41.3% for chronic obstructive pulmonary disease [11]. Such uncertainty of CVD progression and sudden death aggravates patients and clinicians as well. In light of this, a recent study integrates age, GDF15 biomarker, and clinical parameters as the ABC death risk score for clinical evaluation of patient safety and decision tree [12]. There has been increasing interest in identifying the novel death risk score model to effectively predict poor prognosis in CKD patients [13,14,15]. Emerging evidence has shown that the prognostic questionnaires of mortality in CKD and specifically in MHD patients serve as useful risk prediction tools for the early detection of clinical events. Currently, a series of practical questionnaires exist, including SARC-F (sluggishness, assistance in walking, rising from a chair, climb stairs, falls), KDQOL (Kidney Disease Quality of Life) instrument, Karnofsky Performance Status Scale, and 36-Item Short Form Survey (SF-36) [16]. In addition to the above prognostic questionnaires of mortality, a novel biomarker-based risk score system could be developed as an alternative screening tool. With regard to huge prevalence of CV morbidity and mortality in MHD populations, we aimed to assess the predictive validity of GDF15-based risk scores to develop a reliable early warning system for timely management of clinical events. Therefore, various risk factors for all-cause and CVD-related mortality in MHD patients were estimated using a Cox regression model and receiver operating characteristic (ROC) analysis in the present study.

## 2. Materials and Methods

### 2.1. Participants in the Cohort

The study had been approved by the Research Ethics Review Committee of En Chu Kong Hospital (ECKIRB1070102) in accordance with the ethical standards of the committee and the Helsinki declaration for research in humans. The written informed consent was obtained from the participants of this study. The relevant details of the research methods were described previously [1,3]. Patients undergoing MHD treatment for at least three months were eligible for inclusion. All patients had to be older than 18 years of age and be receiving thrice-weekly MHD, and 188 patients in end-stage kidney disease were included. Eighteen patients were excluded from the study because of inadequate dialysis, terminal illness, active infections, advanced cancer, active hepatitis, severe protein-energy wasting, incomplete data, or unwilling to participate.

### 2.2. Assessment of Exposures

The plasma GDF15 and bio-clinical parameters were ascertained at baseline. Plasma concentrations of GDF15 were measured by a commercial quantitative enzyme-linked immunosorbent (ELISA) assay (Human GDF15 ELISA Kit (Catalog Number: DGD15), R&D Systems, Minneapolis, MN, USA) in accordance with the manufacturers’ instructions. Plasma GDF15 concentrations were categorized into tertiles: low, medium, and high levels for tertiles 1, 2, and 3, respectively. In the primary analyses, we examined the association between GDF15 levels, relevant clinical parameters, all-cause, and CV mortality in the Cox regression model. The predictive value of GDF15 tertiles for all-cause and CV mortality was examined using the ROC analysis. In the secondary analyses, we used a simple scale system to determine GDF15-based death risk score: GDF15 tertiles 1 as score 1, tertiles 2 as score 2, and tertiles 3 as score 3. To strengthen the predictive value of GDF15-based score system, the significant clinical predictors in primary analyses were combined with GDF15 score as a GDF15-based death prediction model. The score (1 or 0) of the clinical predictors was defined by the cut-off value in the ROC analysis. All patients were then stratified by the GDF15-based risk score system into new categories. The predictive value of GDF15-based risk score system for all-cause and CV mortality was examined using the ROC analysis.

### 2.3. Assessment of Covariates

The following bio-demographic and laboratory parameters of each patient were recorded at baseline: age, gender, hypertension, diabetes mellitus (DM), previous CVD, hemodialysis (HD) vintage, systolic blood pressure, diastolic blood pressure, pre-dialysis blood urea nitrogen, normalized protein catabolic rate, creatinine, potassium, calcium, phosphorus, alkaline phosphatase (ALP), alanine aminotransferase, albumin, uric acid, total cholesterol, triglyceride, hemoglobin, platelet, and intact parathyroid hormone (iPTH). Previous CV diseases (CVD) were defined as diseases attributable to myocardial ischemia and infarction (ICD-10-CM Diagnosis Code: I20-I25), heart failure (ICD-10-CM Diagnosis Code: I50.1-I50.9), symptomatic or life-threatening arrhythmia (ICD-10-CM Diagnosis Code: I49), cerebrovascular diseases (ICD-10-CM Diagnosis Code: I60-I69), pulmonary embolism (ICD-10-CM Diagnosis Code: I26), and peripheral artery diseases (ICD-10-CM Diagnosis Code: I73). The HD vintage was defined as the duration of time between the first day of HD treatment and the first day that the patient entered the cohort. The blood pressure was recorded in the horizontal recumbent position before a midweek dialysis session. Pre-dialysis blood samples were obtained from the existing vascular access for the further analyses. We adjusted plasma calcium according to the following equation: adjusted calcium, measured calcium + ((4.0-plasma albumin in g/dL) × 0.8). All laboratory tests were performed by the standard procedures with certified methods.

### 2.4. Ascertainment of Outcomes

CV mortality in study patients was defined as death attributable to myocardial ischemia and infarction, heart failure, fatal arrhythmia, cardiac arrest because of other causes, cerebrovascular diseases, pulmonary embolism, peripheral artery diseases, and sudden otherwise unexplained death. Non-CV mortality was defined as all other causes of death, i.e., infection, malignancies, gastrointestinal hemorrhage, accidents, and miscellaneous. All-cause mortality included CV and non-CV death. Patients were censored at last follow-up, switched to another dialysis unit, or received a kidney transplant.

### 2.5. Statistical Analysis

The continuous variables were presented as mean ± standard deviation, and the categorical variables were expressed as number (%). The correlation coefficients between covariates of interest were calculated. The univariate Cox regression analysis was performed to investigate the independence of risk factors associated with all-cause and CV mortality. If hazard ratios (HRs) for the variables in the univariate analysis were significant, we would select the covariates into the multivariable regression model and GDF15-based death prediction model. Multivariable adjusted hazard ratios (aHRs) of different mortality risks were calculated for plasma GDF15 tertiles in the Cox regression model. The cumulative survival probability and proportional hazards were presented by graphical methods. To assess the predictive accuracy of GDF15 tertiles and GDF15-based risk score for mortality, the area under ROC curve (AUC) was used as the criterion [3]. An AUC of 0.5 indicates no predictive ability, whereas a value of 1 represents perfect predictive ability. A *p*-value < 0.05 was considered statistically significant. We used the PASW Statistics SPSS version 22.0 (IBM, City, NY, USA) to analyze all bio-clinical data of MHD patients.

## 3. Results

### 3.1. Primary Analyses: Evaluation of the Clinical Candidate Predictors

The final study sample included 170 MHD patients with complete medical records and follow-up. Baseline bio-clinical data of the whole study population with comparisons between different concentration groups are summarized in Table 1. Plasma GDF15 levels were categorized into low levels (<1314.8 pg/mL), medium levels (1314.8–1707.1 pg/mL), and high levels (>1707.1 pg/mL) as the tertiles 1, 2, and 3, respectively. Age, prevalence of DM, prior history of CVD, HD vintage, and albumin levels were significantly different among three GDF15 tertiles. The mean age of low, medium, and high tertile was 59.1 ± 6.8, 60.3 ± 8.1, and 71.1 ± 7.1 years; namely, the older adults exhibit higher GDF15 levels. The prevalence of DM was 41.1%, 42.9%, and 50.0% in tertiles 1, 2 and 3, respectively. Patients in tertile 3 had the highest prevalence of a prior CVD history (62.5%). Patients in tertile 3 had the longest HD vintage (80.9 ± 34.2 months). The mean albumin level in the tertiles 1, 2 and 3 was 4.0 ± 0.4, 3.9 ± 0.4 and 3.8 ± 0.5 g/dL, respectively. In the univariate Cox regression analysis of clinical prognostic factors (Table 2), older age, higher GDF15 and lower albumin levels were significantly associated with all-cause mortality (HR: 1.074 (95% confidence interval (CI): 1.037–1.112), *p* < 0.01; 1.001 (95% CI: 1.000–1.001), *p* < 0.01; 0.200 (95% CI: 0.100–0.402), *p* < 0.01; respectively). Furthermore, the associations between age, higher GDF15, and lower albumin levels for CV mortality remained significant [HR: 1.086 (95% CI: 1.040–1.133), *p* < 0.01; 1.001 (95% CI: 1.000–1.001), *p* < 0.01; 0.377 (95% CI: 0.160–0.884), *p* < 0.05; respectively). Although the HD vintage was associated with all-cause mortality (HR: 1.006 (95% CI: 1.001–1.011), *p* < 0.05), the association between HD vintage and CV mortality was insignificant [HR: 1.004 (95% CI: 0.998–1.011), *p* = 0.17]. Collectively, patients with older age, higher GDF15 and lower albumin levels are more likely to have greater risks in all-cause and CV mortality. Table 3 summarizes the multivariate Cox regression analysis of clinical candidate predictors for all-cause and CV mortality. Age, GDF15, and albumin were independently associated with overall mortality (aHR: 1.044 (95% CI: 1.007–1.083), *p* < 0.05; 1.001 (95% CI: 1.000–1.001), *p* < 0.01; 0.281 (95% CI: 0.141–0.560), *p* < 0.01; respectively).

**Figure 1:**
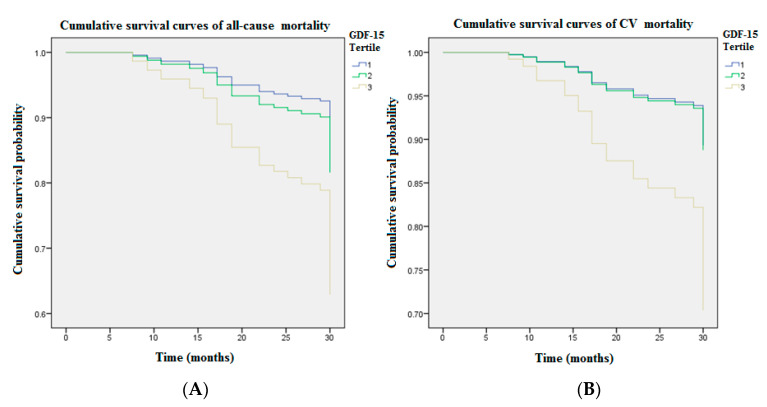
The cumulative survival curves of mortality risks showed GDF15 tertiles remained significant after adjusting for age and albumin during 4650.9 person-months follow-up (Tertile 1 GDF15 < 1314.8 pg/mL; Tertile 2 GDF15 = 1314.8–1707.1 pg/mL; Tertile 3 GDF15 > 1707.1 pg/mL). (**A**) The highest tertile of GDF15 (>1707.1 pg/mL) was associated with an incremental risk of all-cause mortality (aHR: 3.06 [95% CI: 1.20–7.82], *p* < 0.05). (**B**) The association between the highest tertile of GDF15 and CV mortality remained robust after multivariable adjustment (aHR: 3.11 [95% CI: 1.02–9.50], *p* < 0.05). GDF15 = growth differentiation factor-15.

**Figure 2:**
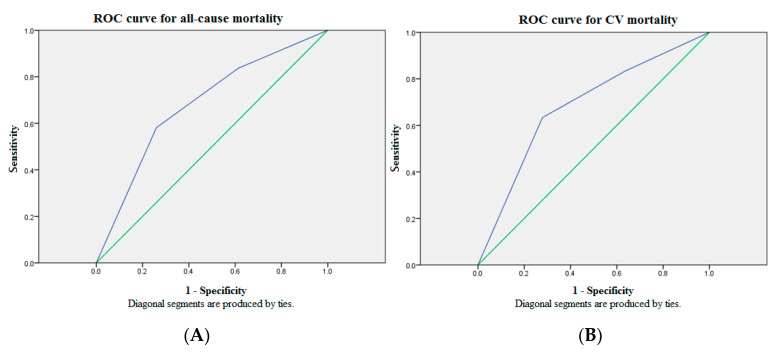
The ROC analysis of GDF15 tertiles for mortality showed suboptimal discriminatory power in the whole study population over 30 months of follow-up. (**A**) The AUC provided by GDF15 tertiles for all-cause mortality was 0.681 (95% CI = 0.589 to 0.774, *p* < 0.01). (**B**) The AUC provided by GDF15 tertiles for CV mortality was 0.684 (95% CI = 0.577 to 0.791, *p* < 0.01). Source of the curve: green line = GDF15 tertiles; blue line = reference. AUC = area under curve; CV = cardiovascular; GDF15 = growth differentiation factor-15; ROC = receiver operating characteristics.

Figure 1 illustrates cumulative survival curves of mortality risks among patients with three GDF15 concentration tertiles after adjusting for age and albumin. The highest tertile of GDF15 is associated with an incremental risk of all-cause mortality (aHR: 3.06 [95% CI: 1.20–7.82], *p* < 0.05). The association between the highest tertile of GDF15 and CV mortality remains robust after multivariable adjustment (aHR: 3.11 [95% CI: 1.02–9.50], *p* < 0.05). Figure 2 demonstrated the ROC analysis using GDF15 tertiles as a predictor of mortality in the whole study population following up for over 30 months. The AUC provided by the tertiles of GDF15 for all-cause and CV mortality is 0.681 (95% CI = 0.589 to 0.774, *p* < 0.01) and 0.684 (95% CI = 0.577 to 0.791, *p* < 0.01), respectively. The ROC analysis using age as a predictor of overall mortality is demonstrated in the Figure 1. The AUC provided by older age for overall mortality is 0.687 (95% CI = 0.594 to 0.779, *p* < 0.01). The cut-off value for age is 65 years old. The ROC analysis using the lower albumin level as a predictor of overall mortality is demonstrated in the Figure 2. The AUC provided by the lower albumin level for overall mortality is 0.691 (95% CI = 0.600 to 0.782, *p* < 0.01). The cut-off value for the albumin level is 3.8 g/dL. As a result, age > 65 years old (score 1), GDF15 tertiles 1–3 (score 1–3) and hypoalbuminemia < 3.8 g/dL (score 1) were combined as a death score system in the secondary analyses.

### 3.2. Secondary Analyses: Evaluation of the GDF15-Based Death Prediction Model

**Figure 3:**
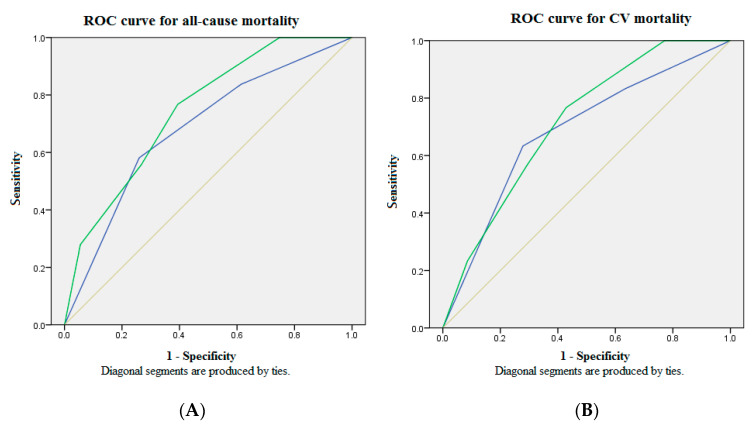
The ROC analysis of the GDF15-based risk score model showed optimal discriminatory power with comparison to GDF15 tertiles in the whole study population over 30 months of follow-up. (**A**) The AUC provided by GDF15 tertiles and the GDF15-based risk score model for all-cause mortality was 0.681 (95% CI = 0.589 to 0.774) and 0.745 (95% CI = 0.666 to 0.824), respectively. (**B**) The AUC provided by GDF15 tertiles and the GDF15-based risk score model for CV mortality was 0.684 (95% CI = 0.577 to 0.791) and 0.715 (95% CI = 0.625 to 0.805), respectively. Source of the curve: green line = GDF15-based risk score model; blue line = GDF15 tertiles; yellow line = reference. AUC = area under curve; CV = cardiovascular; GDF15 = growth differentiation factor-15; ROC = receiver operating characteristics.

Table 4 summarizes the bio-clinical data and fatal events according to our GDF15-based risk score system. Three GDF15 concentration tertiles are designated as scores 1, 2 and 3. Scores 1 or 0 of the clinical predictors are defined by the cut-off value of the ROC analysis. All patients are stratified by the GDF15-based risk score system into new categories. Figure 3 shows the comparison of ROC analysis between GDF15 tertiles and the GDF15-based risk score model for mortality in the whole study population over 30 months of follow-up. The AUC provided by the GDF15-based risk score model for all-cause mortality is 0.745 (95% CI = 0.666 to 0.824), and CV mortality 0.715 (95% CI = 0.625 to 0.805). Consideration of GDF15 concentration in the risk factor analysis increases the sensitivity and the specificity for all-cause and CV mortality. In conclusion, our GDF15-based risk score system may serve as an early warning for timely and appropriate intervention of clinical events in the MHD population.

## 4. Discussion

The optimal medical services to improve the patient safety are delivery of early detection and sustained monitoring of high-risk subjects. To deploy a timely and effective therapeutic strategy in preparation, a robust surrogate biomarker to warn clinicians in advance is crucial for the CKD population because of high prevalence of morbidity and mortality. Especially in MHD patients prone to sudden cardiac death due to persistent inflammation accompanied by malnutrition, the precise prognostic evaluation and therapeutic decision-making are particularly important [15]. Considering their underlying multiple comorbidities, it seems unlikely that a single biomarker assessment is sufficient to understand patients’ overall burden of ailments. Hijazi et al. consolidated age, biomarkers, and clinical parameters as a novel ABC-death risk score in the high-risk CV population [17]. Based on their ABC-death risk model, all clinical candidate predictors in our MHD patients were evaluated using the Cox regression analysis for all-cause and CV mortality in the first step (Table 2). Our results demonstrate MHD patients with older age, higher plasma GDF15 concentrations, and lower albumin levels are more likely to have greater risks for all-cause death (HR: 1.074 ((95% CI: 1.037–1.112), 1.001 ((95% CI: 1.000–1.001), 0.200 (95% CI: 0.100–0.402), respectively) but also CV mortality (HR: 1.086 (95% CI: 1.040–1.133), 1.001 (95% CI: 1.000–1.001), 0.377 (95% CI: 0.160–0.884), respectively). HD vintage lost the predictive power for CV mortality (Table 2), despite being associated with all-cause death and significantly different among three groups of GDF15 tertiles (Table 1). In the multivariate Cox regression analysis for clinical candidate predictors (Table 3), age, GDF15, and albumin were independently associated with overall mortality (aHR: 1.044 (95% CI: 1.007–1.083), 1.001 (95% CI: 1.000–1.001), 0.281 (95% CI: 0.141–0.560), respectively). Accordingly, older age, higher GDF15, and hypoalbuminemia were integrated as a novel death risk score system in our modified prediction model. After adjusting for age and albumin in the cumulative survival curve analysis with respect to different GDF15 tertiles among the whole study population (Figure 1), the highest tertile of GDF15 (>1707.1 pg/mL) was associated with an incremental risk for not only all-cause death (aHR: 3.06 (95% CI: 1.20–7.82), *p* < 0.05) but also CV mortality (aHR: 3.11 (95% CI: 1.02–9.50), *p* < 0.05). In the ROC analysis for mortality over 4650.9 person-months follow-up, however, the AUC provided by GDF15 tertiles for all-cause and CV mortality was 0.681 (95% CI = 0.589 to 0.774, *p* < 0.01) and 0.684 (95% CI = 0.577 to 0.791, *p* < 0.01), respectively. The prediction model using GDF15 tertiles has inadequate discriminatory power (Figure 2). Likewise, the AUC provided by older age (>65 years old) for overall mortality is 0.687 (95% CI = 0.594 to 0.779, *p* < 0.01) (Figure 1). The AUC provided by the lower albumin level (<3.8 g/dL) for overall mortality is 0.691 (95% CI = 0.600 to 0.782, *p* < 0.01) (Figure 2). The above results inspire us to combine age, GDF15, and hypoalbuminemia as a brand-new risk score system to predict fatal events.

In the secondary analyses, age, biomarker GDF15, and hypoalbuminemia were incorporated into a novel GDF15-baesd death prediction model for the ROC analysis. As expected, our GDF15-based risk score is a more comprehensive system that provides stronger predictive values for both all-cause and CV mortality. The comparison of ROC analyses between GDF15 tertiles and the GDF15-based risk score model for mortality was provided in Figure 3. The AUC provided by the GDF15-based risk score model for all-cause and CV mortality was 0.745 (95% CI = 0.666 to 0.824) and 0.715 (95% CI = 0.625 to 0.805), respectively.

GDF15 is reminiscent of a robust prognostic biomarker in the high-risk patients with CVD, including fatal arrhythmia, myocardial infarction and heart failure [17,18,19,20]. In addition to the predictive role of cardiac biomarker, increased GDF15 levels have been known to be associated with a greater risk of all-cause mortality [21]. However, there are few studies linking GDF15 to the CKD-related outcome-prediction models. Previous studies demonstrated that a higher circulating level of GDF15 was an independent predictor of CKD mortality after multivariate adjustment, and correlated with the severity of atherosclerosis, hypoalbuminemia, inflammation, HD vintage, and protein-energy wasting [22,23]. Nair et al. found that plasma GDF15 levels were correlated with intrarenal GDF15 expression in the tubulo-interstitial compartment and significantly associated with a decline in renal function or progression to end stage renal disease. Their research team concluded that GDF15 was not only a CKD outcome predictor, but rather directly involved in the intrarenal signaling pathways contributing to CKD progression [6]. Bansal et al. analyzed the baseline characteristics of 3664 CKD patients and found that those with higher plasma GDF15 concentrations had older age, the higher prevalence of DM and CVD history, and higher risks in CKD progression [8]. The results above support our findings that higher GDF15 levels were associated older age, longer HD duration, hypoalbuminemia, and higher risks in the fatal events. We acknowledge that the reliability of our ABC-death score model might be insufficient in relation to the relatively small sample size; however, such ABC-death prediction model was validated in a large sample study of 14,611 patients [12]. Furthermore, You et al. measured GDF15 using the same method and enzyme-linked immunosorbent assay kits (R&D Systems, Minneapolis, MN, USA) as we used [10]. Their main result is similar to ours in which GDF15 is associated with all-cause mortality using adjusted death HRs. Both their and our study subjects are hemodialysis patients, suggesting that the patient heterogeneity may not exert a significant influence. Considering the nature of GDF15 is a stress-induced protein, elevated circulating GDF15 levels indeed assisted physicians in identifying the patients at a high risk for a myriad of adverse clinical outcomes.

Our study contributes differently from other mortality risk indicators based on a previously validated ABC-death risk score model in a large sample size of 14,611 high-risk CV patients [12]. We confirmed the external validity and generalizability of the ABC-death prediction model in the MHD population. Furthermore, we proved that a combination scoring system provides more robust predictive powers than a single mortality risk indicator. Considering the multiple comorbidities in patients with organ failure, it seems unlikely that a single biomarker assessment could comprehensively evaluate the patients’ overall disease burden. Thus, we improve the insufficiency of single mortality risk indicator. In light of this, our risk scoring system could be applied in the monthly routine assessment of MHD patients. An elevated GDF15-based risk score warns clinicians to determine an appropriate intervention in advance.

The interest is increasing rapidly in the application of risk score systems and artificial intelligence (AI)-based decision tree assisted technologies to monitor the patient safety [14,15]. In recent years, the accessibility of electronic medical records for big data in the healthcare and development of machine learning to AI has grown tremendously. Using machine-learning algorithms to analyze a huge amount of clinical data/risk factors in the patient outcome prediction has been demonstrated to be effective [23,24] and, in some case, even surpassing the performance of clinicians [25,26]. Our GDF15-based risk score system is designed based on the statistic regression analysis of clinical parameters and a robust biomarker to predict the mortality of CKD patients, providing a data bank in AI development. With the calibration settings of AI algorithms and extensive validation in the future, we hope this model can be implemented in clinical practice to alarm clinicians and patients for deploying a therapeutic strategy and decision-making in advance.

Several limitations of this study are noteworthy. Firstly, our study sample size was relatively small and the majority of study participants were Asian. Thus, our model applied to other populations should be interpreted with caution. Secondly, cross-sectional laboratory values and GDF15 levels might not reflect substantial intra-individual variability over time. Finally, all cases of sudden death and CV events were combined as CV mortality because we lacked sufficient power to examine specific events. We acknowledge that the pathophysiology of underlying CVD for various outcomes may differ.

## 5. Conclusions

Since the risk stratification alerts clinicians to deploy a differential diagnosis and corresponding treatment in advance, searching for a robust biomarker-based risk prediction model is of prime importance for MHD patients with extremely high mortality rates. Age, GDF15, and hypoalbuminemia predict all-cause and CV death in MHD patients, yet a combination scoring system provides more robust predictive powers. Elevated GDF15-based risk score serves as a warning sign, indicating an urgent need for the further study to determine an appropriate intervention. In light of this, the GDF15-based death prediction model should be developed in the AI-based precision medicine.

## Abbreviation

aHRs adjusted hazard ratiosAI artificial intelligenceALTAlanine aminotransferaseAST aspartate aminotransferaseAUC area under ROC curveBUN blood urea nitrogenCI confidence intervalCKD chronic kidney diseaseCa-P Calcium-phosphateCV cardiovascularCVD cardiovascular diseasesDBP Diastolic blood pressureDM Diabetes mellitusGDF15 growth differentiation factor-15HD HemodialysisiPTH intact parathyroid hormoneLDL Low-density lipoproteinMHD maintenance hemodialysisnPCRnormalized protein catabolic rateROC receiver operating characteristicSBP Systolic blood pressureT-Cholesterol Total Cholesterol

## Figures and Tables

**Table 1:** Age, diabetes mellitus, cardiovascular diseases, hemodialysis vintage, and albumin differ significantly in the comparisons of baseline bio-clinical characteristics according to GDF15 tertiles.

Variables	Tertile 1 <1314.8 pg/mL	Tertile 2 1314.8–1707.1 pg/mL	Tertile 3 >1707.1 pg/mL
Patients, *n* (%)	56 (32.9)	56 (32.9)	58 (41.4)
**Age (years)**	**59.1 ± 6.8**	**60.3 ± 8.1**	**71.1 ± 7.1**
Male, *n* (%)	31 (55.4)	27 (48.2)	24 (41.4)
**Diabetes mellitus, *n* (%)**	**23 (41.1)**	**24 (42.9)**	**29 (50.0)**
**Cardiovascular diseases, *n* (%)**	**80 (47.9)**	**51 (40.2)**	**25 (62.5)**
Hypertension, *n* (%)	26 (46.4)	32 (57.1)	34 (58.6)
Systolic blood pressure (mmHg)	135.3 ± 21.4	134.1 ± 21.7	142.3 ± 22.7
Diastolic blood pressure (mmHg)	76.4 ± 8.6	78.3 ± 11.4	79.3 ± 14.2
**Hemodialysis vintage (months)**	**72.2 ± 58.4**	**62.5 ± 51.8**	**80.9 ± 34.2**
nPCR (g/kg/day)	1.2 ± 0.3	1.1 ± 0.3	1.1 ± 0.3
**GDF15 (pg/mL)**	**1046.6 ± 203.0**	**1525.4 ± 112.7**	**2298.6 ± 637.9**
**Albumin (g/dL)**	**4.0 ± 0.4**	**3.9 ± 0.4**	**3.8 ± 0.5**
Aspartate aminotransferase (IU/L)	15.0 ± 5.1	15.5 ± 6.8	17.8 ± 8.4
Alanine aminotransferase (IU/L)	13.2 ± 8.3	14.8 ± 13.6	16.6 ± 13.0
Total cholesterol (mg/dL)	192.2 ± 51.3	184.3 ± 47.6	192.5 ± 46.7
Triglyceride (mg/dL)	243.7 ± 201.4	194.4 ± 172.1	179.8 ± 23.6
Low-density lipoprotein	108.1 ± 39.4	101.9 ± 34.1	111.8 ± 33.5
Blood urea nitrogen (mg/dL)	55.9 ± 17.8	59.5 ± 17.7	61.8 ± 20.0
Creatinine (mg/dL)	9.4 ± 2.0	10.0 ± 1.9	10.0 ± 1.6
Blood glucose (mg/dL)	141.7 ± 59.0	143.3 ± 65.0	135.1 ± 84.5
Uric acid (mg/dL)	7.4 ± 1.4	7.3 ± 1.3	7.2 ± 1.1
Potassium (mmol L^−1^)	4.6 ± 0.9	4.6 ± 0.9	4.3 ± 0.8
Calcium (mg/dL)	9.4 ± 0.8	9.3 ± 0.8	9.1 ± 0.7
Phosphate (mg/dL)	4.6 ± 1.7	4.3 ± 1.4	5.0 ± 1.4
Calcium-phosphate product	42.1 ± 15.1	40.2 ± 12.9	45.9 ± 13.6
Intact parathyroid hormone (pg/mL)	164.1 ± 220.5	153.8 ± 145.2	184.9 ± 200.2
Hemoglobin (g/dL)	10.8 ± 1.3	10.4 ± 1.2	10.6 ± 1.3
Hematocrit (%)	32.2 ± 3.8	31.1 ± 3.5	32.0 ± 3.7
Platelet (k/μL)	209.3 ± 66.3	185.5 ± 71.4	199.9 ± 56.1

Continuous variables were expressed as mean ± SD. Categorical variables are expressed as *n* (%). Boldface indicates where the values differ significantly between GDF15 tertiles. GDF15 = growth differentiation factor-15. nPCR = normalized protein catabolic rate.

**Table 2:** Age, GDF15, and albumin as significant prognostic parameters for all-cause and CV mortality in the univariate Cox regression analysis.

	All-Cause Mortality		CV Mortality	
	HR (95% CI)	*p*-Value	HR (95% CI)	*p*-Value
**Age**	**1.074 (1.037–1.112)**	***p* < 0.01**	**1.086 (1.040–1.133)**	***p* < 0.01**
Male	0.867 (0.476–1.578)	*p* = 0.64	1.139 (0.556–2.335)	*p* = 0.72
HD vintage	1.006 (1.001–1.011)	*p* < 0.05	1.004 (0.998–1.011)	*p* = 0.17
**GDF15**	**1.001 (1.000–1.001)**	***p* < 0.01**	**1.001 (1.000–1.001)**	***p* < 0.01**
SBP	1.012 (0.998–1.026)	*p* = 0.08	1.021 (1.005–1.038)	*p* < 0.05
DBP	0.981 (0.998–1.026)	*p* = 0.16	0.982 (0.952–1.014)	*p* = 0.27
Blood glucose	1.001 (0.996–1.006)	*p* = 0.72	1.003 (0.998–1.008)	*p* = 0.30
nPCR	0.840 (0.284–2.488)	*p* = 0.75	0.875 (0.239–3.200)	*p* = 0.84
**Albumin**	**0.200 (0.100–0.402)**	***p* < 0.01**	**0.377 (0.160–0.884)**	***p* < 0.05**
AST	0.983 (0.938–1.030)	*p* = 0.47	0.957 (0.898–1.019)	*p* = 0.17
ALT	1.014 (0.993–1.036)	*p* = 0.19	1.018 (0.994–1.043)	*p* = 0.14
T-Cholesterol	0.999 (0.992–1.005)	*p* = 0.65	1.000 (0.993–1.008)	*p* = 0.90
Triglyceride	0.998 (0.996–1.001)	*p* = 0.15	0.998 (0.995–1.001)	*p* = 0.19
LDL	0.998 (0.990–1.006)	*p* = 0.63	1.001 (0.991–1.011)	*p* = 0.83
BUN	1.008 (0.992–1.023)	*p* = 0.34	1.009 (0.991–1.028)	*p* = 0.34
Creatinine	1.029 (0.879–1.204)	*p* = 0.73	1.113 (0.920–1.347)	*p* = 0.27
Uric acid	1.077 (0.859–1.350)	*p* = 0.52	1.088 (0.830–1.426)	*p* = 0.54
Potassium	0.788 (0.552–1.125)	*p* = 0.19	0.773 (0.504–1.185)	*p* = 0.24
Calcium	0.795 (0.515–1.229)	*p* = 0.30	0.602 (0.347–1.045)	*p* = 0.07
Phosphate	1.075 (0.897–1.288)	*p* = 0.44	1.038 (0.829–1.300)	*p* = 0.75
Ca-P product	1.005 (0.984–1.026)	*p* = 0.64	0.999 (0.973–1.025)	*p* = 0.92
iPTH	1.001 (1.000–1.002)	*p* = 0.07	1.001 (0.999–1.002)	*p* = 0.43
Hemoglobin	1.066 (0.818–1.390)	*p* = 0.63	1.013 (0.739–1.389)	*p* = 0.94
Hematocrit	1.032 (0.939–1.133)	*p* = 0.51	1.017 (0.910–1.137)	*p* = 0.77
Platelet	1.002 (0.998–1.007)	*p* = 0.35	1.005 (0.997–1.010)	*p* = 0.09

Boldface indicates significant predictors for both all-cause and CV mortality. ALT = Alanine aminotransferase; AST = aspartate aminotransferase; BUN = blood urea nitrogen; Ca-*p* = Calcium-phosphate; CV = cardiovascular; CVD = cardiovascular diseases; CI = confidence interval; GDF15 = growth differentiation factor-15; HD = Hemodialysis; HR, hazard ratio; iPTH = intact parathyroid hormone; LDL = Low-density lipoprotein; nPCR = normalized protein catabolic rate; SBP = Systolic blood pressure; T-Cholesterol = Total Cholesterol.

**Table 3:** Age, GDF15, and albumin as significant prognostic parameters for all-cause mortality in the multivariate Cox regression analysis.

	All-Cause Mortality		CV Mortality	
	HR (95% CI)	*p*-Value	HR (95% CI)	*p*-Value
Age	1.044 (1.007–1.083)	*p* < 0.05	1.062 (1.015–1.112)	*p* < 0.01
GDF15	1.001 (1.000–1.001)	*p* < 0.01	1.001 (1.000–1.001)	*p* < 0.05
Albumin	0.281 (0.141–0.560)	*p* < 0.01	0.550 (0.235–1.289)	*p* = 0.17

CVD = cardiovascular diseases; CI = confidence interval; GDF15 = growth differentiation factor-15.

**Table 4:** Comparison of bio-clinical parameters and fatal events according to GDF15-based risk score system in the whole study population.

	Score = 1	Score = 2	Score = 3	Score = 4	Score = 5
Patients, *n* (%)	32 (18.8)	55 (32.4)	25 (14.7)	39 (22.9)	19 (11.2)
All-cause death, *n* (%)	0 (0)	10 (18.2)	9 (36.0)	12 (30.8)	12 (63.2)
CV death, *n* (%)	0 (0)	7 (12.7)	6 (24.0)	10 (25.6)	7 (36.8)
Age (years)	56.3 ± 4.0	58.9 ± 6.4	61.6 ± 7.1	71.2 ± 6.6	74.2 ± 4.7
Male, *n* (%)	12 (37.5)	33 (60.0)	15 (60.0)	13 (33.3)	9 (47.4)
DM, *n* (%)	9 (28.1)	24 (43.6)	12 (48.0)	20 (51.3)	11 (57.9)
CVD, *n* (%)	12 (37.5)	22 (40.0)	11 (44.0)	24 (61.5)	10 (52.6)
Hypertension, *n* (%)	19 (59.4)	29 (52.7)	13 (52.0)	21 (53.8)	10 (52.6)
SBP (mmHg)	136.4 ± 16.4	134.3 ± 23.2	135.0 ± 25.5	141.1 ± 21.8	143.8 ± 23.7
DBP (mmHg)	79.1 ± 6.9	79.0 ± 10.7	71.2 ± 11.2	79.7 ± 14.4	79.0 ± 13.1
HD vintage (months)	72.9 ± 49.2	59.6 ± 51.9	81.9 ± 61.6	83.1 ± 41.5	70.4 ± 32.4
nPCR (g/kg/day)	1.2 ± 0.3	1.1 ± 0.3	1.1 ± 0.3	1.0 ± 0.2	1.1 ± 0.4
GDF15 (pg/mL)	1098.3 ± 181.3	1303.8 ± 324.3	1731.8 ± 638.1	2157.2 ± 633.8	2266.7 ± 592.8
Albumin (g/dL)	4.1 ± 0.3	4.0 ± 0.4	3.8 ± 0.4	3.8 ± 0.4	3.3 ± 0.4
AST (IU/L)	15.1 ± 5.0	14.7 ± 5.5	15.7 ± 6.9	18.9 ± 9.1	17.3 ± 8.0
ALT (IU/L)	14.9 ± 8.4	14.1 ± 13.5	13.2 ± 10.6	14.8 ± 11.0	19.3 ± 15.3
T-Cholesterol (mg/dL)	188.5 ± 48.5	190.3 ± 51.3	186.6 ± 43.1	199.4 ± 52.9	186.0 ± 41.6
Triglyceride (mg/dL)	225.7 ± 180.7	222.7 ± 203.9	198.5 ± 165.0	202.1 ± 197.5	172.6 ± 141.6
LDL	107.2 ± 41.4	105.8 ± 36.3	103.0 ± 29.8	114.4 ± 37.4	103.5 ± 28.5
BUN (mg/dL)	53.5 ± 16.1	57.2 ± 17.1	65.1 ± 16.9	59.4 ± 18.0	65.5 ± 25.8
Creatinine (mg/dL)	9.6 ± 2.0	9.8 ± 2.2	10.5 ± 1.4	9.5 ± 1.5	9.9 ± 1.7
Blood glucose (mg/dL)	122.0 ± 40.2	130.9 ± 57.5	152.6 ± 63.2	133.6 ± 70.2	137.0 ± 69.7
Uric acid (mg/dL)	7.5 ± 1.3	7.3 ± 1.5	7.6 ± 1.1	6.9 ± 1.1	7.2 ± 0.9
Potassium (mmol L^-1^)	4.6 ± 1.0	4.5 ± 0.9	4.8 ± 0.7	4.2 ± 0.6	4.1 ± 1.0
Calcium (mg/dL)	9.3 ± 0.7	9.2 ± 0.7	9.4 ± 0.8	9.2 ± 0.7	9.1 ± 0.7
Phosphate (mg/dL)	4.5 ± 1.5	4.4 ± 1.7	5.1 ± 1.5	4.7 ± 1.2	5.0 ± 1.7
Ca-P product	41.6 ± 14.0	40.9 ± 15.2	47.0 ± 14.4	42.8 ± 10.4	44.5 ± 16.2
iPTH (pg/mL)	213.9 ± 254.0	177.5 ± 195.8	328.8 ± 320.2	236.4 ± 230.5	198.4 ± 214.2
Hemoglobin (g/dL)	10.8 ± 1.3	10.6 ± 1.3	10.7 ± 1.1	10.6 ± 1.2	10.4 ± 1.6
Hematocrit (%)	32.0 ± 3.7	31.4 ± 3.7	31.9 ± 3.2	31.9 ± 3.5	31.7 ± 4.5
Platelet (k/μL)	205.2 ± 62.6	200.0 ± 68.2	192.1 ± 78.7	190.4 ± 57.9	203.7 ± 64.1

Continuous variables were expressed as mean ± SD. Categorical variables are expressed as *n* (%). ALT= Alanine aminotransferase; AST = aspartate aminotransferase; BUN = blood urea nitrogen; Ca-P = Calcium-phosphate; CV = cardiovascular; CVD = cardiovascular diseases; DBP = Diastolic blood pressure; DM = Diabetes mellitus; GDF15 = growth differentiation factor-15; HD = Hemodialysis; iPTH = intact parathyroid hormone; LDL = Low-density lipoprotein; nPCR= normalized protein catabolic rate; SBP = Systolic blood pressure; T-Cholesterol = Total Cholesterol.

## Data Availability

The numeric data used to support the findings of this study are available from the corresponding author, Jian-Chiun Liou, upon reasonable request.
